# Impact of body mass index on robotic transaxillary thyroidectomy

**DOI:** 10.1038/s41598-019-45355-0

**Published:** 2019-06-20

**Authors:** Zeng Yap, Won Woong Kim, Sang-Wook Kang, Cho Rok Lee, Jandee Lee, Jong Ju Jeong, Kee-Hyun Nam, Woong Youn Chung

**Affiliations:** 1 Department of Surgery, Yonsei University College of Medicine, Seodaemun-gu, Seoul 03722 South Korea; 2 Asan Medical Center, University of Ulsan College of Medicine, Songpa-gu, Seoul 05505 South Korea

**Keywords:** Adrenal gland diseases, Thyroid diseases

## Abstract

Obesity is associated with increased operating times and higher complication rates in many types of surgery. Its impact on robotic thyroidectomy however, is not well documented. The aim of this study was to investigate the relationship between body mass index (BMI) and robotic transaxillary thyroidectomy (RTAT). A retrospective review of prospectively collected data of all patients who underwent RTAT at Yonsei University Health System from October 2007 to December 2014 was performed. Patients were divided into three groups based on BMI (Group 1: BMI < 25, Group 2: BMI 25–29.99, Group 3: BMI ≥ 30), and compared. A total of 3697 patients were analyzed. No differences between the three groups were observed in clinicopathological factors, extent of surgery or length of stay. After multivariate analysis, only seroma and transient voice hoarseness were related to increasing BMI. Total operative time was significantly longer for Group 3 patients with less-than-bilateral total thyroidectomy (BTT), but was not significantly different for patients with BTT. Although obese patients undergoing RTAT have a slightly higher risk of seroma, transient voice hoarseness, and longer operative times, BMI did not influence the other important surgical outcomes of thyroidectomy. Therefore, obesity should not be a contraindication for performing RTAT.

## Introduction

Minimally invasive surgery (MIS) is an increasingly common technique used in most surgical practices because of advantages of smaller incisions, reduced pain, less blood loss, shorter length of hospital stay, and earlier return to work. MIS for cervical endocrine glands began in 1996 when Gagner performed the first endoscopic subtotal parathyroidectomy in a patient with primary hyperparathyroidism^1^. Subsequently in 1997, Huscher *et al*. described the first endoscopic right thyroid lobectomy^2^. Different endoscopic approaches have since been developed and performed worldwide for better cosmesis and avoidance of neck scars. Endoscopic techniques are however limited by rigid instruments that cannot articulate for more precise retraction and dissection of vital structures around the thyroid gland.

Our institution developed gasless, robotic transaxillary thyroidectomy (RTAT) in 2007^3^. Since then, we have performed more than 5000 thyroidectomies using this technique, achieving results that are comparable in complication rates and oncological outcomes to standard open operation^4^. Some surgeons have, however, been skeptical that such good results are only possible because of favorable patient body habitus, as the Korean population is mostly within the normal BMI range, as opposed to patients from Western countries where the obesity rate is much higher. This difference was highlighted in the 2013–2014 National Health and Nutrition Examination Survey that reported a U.S. obesity rate (BMI ≥ 30 kg/m^2^) of 40.4% in females and 35.0% in males^5^. The obesity rate in South Korea as reported by the Korean National Health and Nutrition Examination Survey for the same period, was 4.3% for females and 5.3% for males^6^.

Despite the significantly lower obesity rate in South Korea, our department still manages a small number of obese patients. Given that few objective data are available on the relationship between obesity and surgical outcomes of patients undergoing RTAT, we, at the largest robotic thyroidectomy center in the world, investigated the impact of BMI on operative time, morbidity and long-term outcomes of RTAT at our institution.

## Materials and Methods

### Patients

A retrospective review of our database containing prospectively collected data of all patients who underwent RTAT at Yonsei University Health System, Seoul, Korea from October 2007 to December 2014 was performed. Patients who had undergone simultaneous lateral neck dissection, completion total thyroidectomy or re-operation were excluded. Patients were divided into three groups based on World Health Organization classification^7^: BMI < 25 kg/m^2^ (normal or underweight, Group 1), BMI 25–29.99 kg/m^2^ (overweight, Group 2) and BMI ≥ 30 kg/m^2^ (obese, Group 3). The minimum follow-up period was 30 months. This study was approved by the Institutional Review Board (IRB) of Yonsei University College of Medicine (IRB number 4–2018–0375). Individual patient consent was waived because of the retrospective nature of the study and no patient identifying information was used.

### Surgery

Operations were subdivided into three components: creation of working space (flap dissection), docking (docking of robotic arms), and console times (time to perform thyroidectomy). Operations were defined as BTT, or less-than-BTT if ipsilateral lobectomy was performed with or without partial contralateral thyroidectomy. We routinely performed prophylactic central compartment neck dissection (CCND) for malignant cases. All surgeries were performed by consultant surgeons or training fellows in our hospital.

### Clinicopathological factors

Patients’ clinicopathological factors including age, sex, tumor size, multifocality, bilaterality, extra-capsular invasion, mean number of central nodes harvested, and central node positivity were recorded and compared. Operative times, complications, and recurrences were also analyzed.

### Statistical analysis

Continuous variables are reported as mean ± standard deviation, and comparisons between groups were performed using Student’s t-test. Categorical data are recorded as numbers with percentage and analyzed with Pearson χ^2^ test or Fisher’s exact tests. Associations between surgical outcomes and operative times and clinicopathological factors including BMI were assessed using logistic regression and multiple linear regression. Statistical significance was set at *p* < 0.05. All statistical analyses were performed using SPSS software version 23.0 (IBM Corp., Armonk, NY).

## Results

### Clinicopathological factors

**Figure 1:**
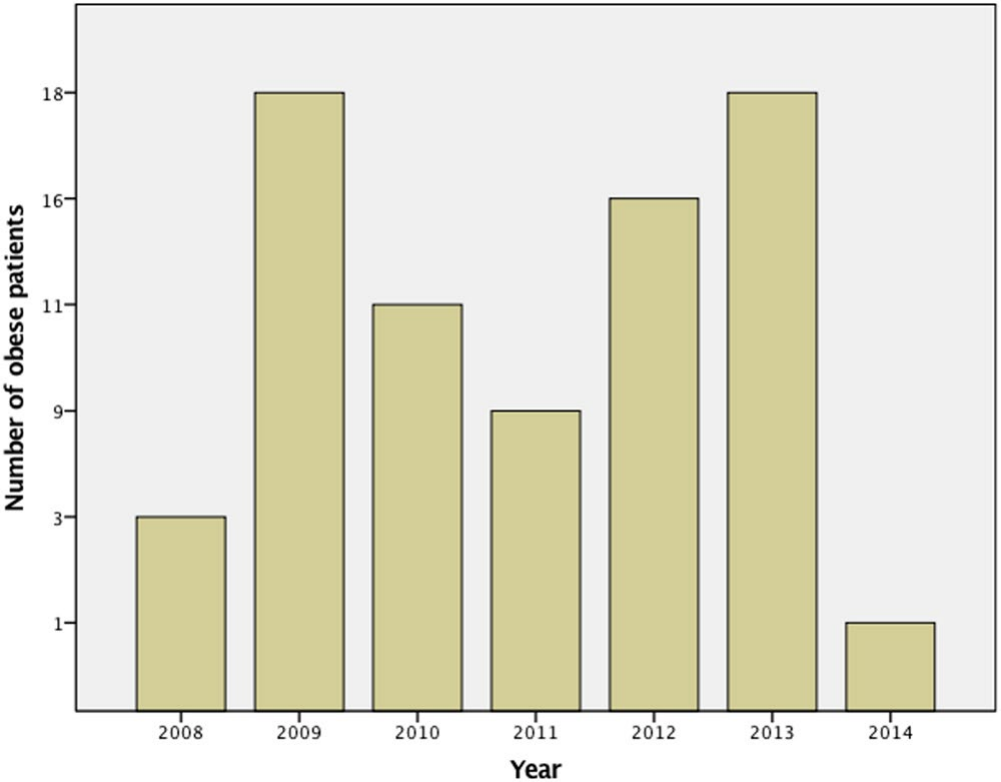
Number of obese patients treated over time.

**Figure 2:**
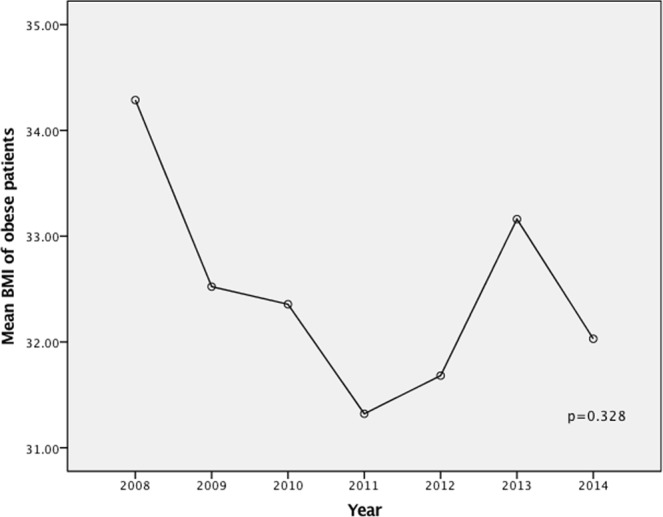
Mean BMI of obese patients according to year of surgery.

A total of 3697 patients were included. Group 1 consisted of 3062 patients, Group 2 had 559 and Group 3 had 76 patients. The mean age of the entire cohort was 38.7 ± 9.2 years, the percentage of females was 89.3% and the percentage of males was 10.7%. The number and mean BMI of obese patients were randomly distributed across the study period (Figs 1 and 2). Mean tumor size was 0.79 ± 0.59 cm, and malignancy rate was 96.2%. Multifocal tumors were found in 22.2% of our patients, and 11.3% of tumors were bilateral. Extracapsular invasion was present in 43.5% of patients. There was no significant difference in the distribution of patients across the three groups according to the TNM staging for malignant patients (Table 1). Most of the cancers were T1 cancers measuring less ≤ 2 cm in size (55.0%), whilst 1% were T2 cancers (2–4 cm). 43.8% of the cases were T3 cancers with minimal extrathyroidal tumor extension, according to the 7^th^ Edition of the TNM Classification System for Differentiated Thyroid Carcinoma^8^. The mean number of central nodes removed was 4.60 ± 3.85, and the mean number of positive nodes was 0.74 ± 1.58. Mean post-operative inpatient stay was 3.23 ± 1.6 days, and recurrence rate was 0.6%. Comparisons among the three groups found no significant differences apart from age, sex, and tumor multifocality (Table 1). Multifocality was no longer significant after multivariate analysis. Recurrence rates were also similar across the three groups, at 0.6% for the entire cohort.

**Table 1:** Patient demographics and clinicopathological factors.

		Total cohort	Group 1 (BMI < 25 kg/m^2^)	Group 2 (BMI 25–29.99 kg/m^2^)	Group 3 (BMI ≥ 30 kg/m^2^)	*p* value
Number of patients		3697 (100%)	3062 (82.8%)	559 (15.1%)	76 (2.1%)	
Age (years)		38.7 ± 9.2	38.2 ± 9.0	41.5 ± 9.2	38.2 ± 10.1	<0.001*
Gender						0.001*
Female		3303 (89.3%)	2825 (92.3%)	422 (75.5%)	56 (73.7%)	
Male		394 (10.7%)	237 (7.7%)	137 (24.5%)	20 (26.3%)	
Mean BMI		22.2 ± 3.1	21.2 ± 2.0	26.7 ± 1.3	32.4 ± 2.4	0.001*
Mean tumor size (cm)		0.79 ± 0.59	0.78 ± 0.59	0.81 ± 0.60	0.85 ± 0.78	0.498
Malignancy		3555 (96.2%)	2943 (96.1%)	538 (96.2%)	74 (97.4%)	0.848
^§^TNM staging						
T	1	1954 (55.0%)	1620 (55.0%)	291 (54.1%)	43 (58.1%)	0.749
	2	36 (1.0%)	33 (1.12%)	2 (0.37%)	1 (1.36%)	
	3	1558 (43.8%)	1284 (43.6%)	244 (45.4%)	30 (40.5%)	
	4	7 (0.2%)	6 (0.20%)	1 (0.19%)	0 (0.0%)	
N	0	2451 (68.9%)	2042 (69.4%)	367 (68.2%)	42 (56.8%)	0.187
	1a	1094 (30.8%)	892 (30.3%)	170 (31.6%)	32 (43.3%)	
	1b	10 (0.3%)	9 (0.31%)	1 (0.19%)	0 (0.0%)	
M	0	3555 (100%)	2943 (100%)	538 (100%)	74 (100%)	
	1	0 (0.0%)	0 (0.0%)	0 (0.0%)	0 (0.0%)	
Stage	I	3036 (85.4%)	2559 (86.9%)	415 (77.1%)	62 (83.8%)	
	II	4 (0.11%)	3 (0.1%)	1 (0.2%)	0 (0.0%)	
	III	511 (14.4%)	378 (12.9%)	121 (22.5%)	12 (16.2%)	
	IVa	4 (0.13%)	3 (0.1%)	1 (0.2%)	0 (0.0%)	
	IVb	0 (0.0%)	0 (0.0%)	0 (0.0%)	0 (0.0%)	
^#^Multifocal tumor						0.006*
No		2825 (77.6%)	2368 (78.5%)	409 (74.5%)	48 (64.0%)	
Yes		808 (22.2%)	641 (21.3%)	140 (25.5%)	27 (36.0%)	
^#^Bilateral tumor						0.359
No		3246 (88.7%)	2698 (89.1%)	482 (87.0%)	66 (88.0%)	
Yes		412 (11.3%)	331 (10.9%)	72 (13.0%)	9 (12.0%)	
^#^Extracapsular invasion						0.854
No		2023 (56.4%)	1680 (56.5%)	299 (55.1%)	44 (59.5%)	
Yes		1563 (43.5%)	1289 (43.4%)	244 (44.9%)	30 (40.5%)	
Extent of surgery						0.199
Less than BTT		2377 (64.3%)	1987 (64.9%)	346 (61.9%)	44 (57.9%)	
BTT		1320 (35.7%)	1075 (35.1%)	213 (38.1%)	32 (42.1%)	
Mean no. of central nodes removed		4.60 ± 3.85	4.60 ± 3.89	4.52 ± 3.61	5.30 ± 3.96	0.262
Mean no. of positive central node		0.74 ± 1.58	0.73 ± 1.58	0.79 ± 1.60	1.03 ± 1.59	0.208
Post-operative inpatient stay (days)		3.23 ± 1.6	3.22 ± 1.7	3.27 ± 0.9	3.23 ± 1.6	0.674
Recurrence		22 (0.6%)	20 (0.7%)	2 (0.4%)	0 (0.0%)	0.559

BMI, body mass index; BTT, bilateral total thyroidectomy; ^§^AJCC 7^th^ Edition/TNM Classification System for Differentiated Thyroid Carcinoma; ^#^Data not available for the entire cohort; *statistically significant at *p* < 0.05.

### Complications

Major complications associated with thyroidectomy are shown in Table 2. The overall recurrent laryngeal nerve injury rate was 0.4%, hematoma requiring surgical intervention 0.1%, and transient hypocalcaemia rate of 37.4% (in BTT group). No significant difference was seen in complication rates between the three BMI groups except for seroma formation and transient voice hoarseness. After multivariate analysis, transient voice hoarseness was significantly associated with Group 3 patients (OR 3.06; *p* = 0.02), bilateral total thyroidectomy (OR 1.87; *p* = 0.02) and longer console time (OR 1.02; *p* < 0.001). The risk of seroma was higher in both the overweight and obese groups (Group 2 OR 4.59; *p* value < 0.001, Group 3 OR 7.24; *p* < 0.001) (Table 3).

**Table 2:** Complication rates for the three BMI groups.

	Total cohort (N = 3697)	Group 1 (BMI < 25) (N = 3062)	Group 2 (BMI 25–29.99) (N = 559)	Group 3 (BMI ≥ 30) (N = 76)	*p* value
RLN injury	14 (0.4%)	12 (0.4%)	2 (0.4%)	0 (0.0%)	0.857
Transient voice hoarseness	88 (2.4%)	69 (2.3%)	14 (2.5%)	5 (6.6%)	0.049*
#Transient hypocalcemia	494 (37.4%)	414 (38.5%)	69 (32.4%)	11 (34.4%)	0.226
Seroma	55 (1.5%)	28 (0.9%)	23 (4.1%)	4 (5.3%)	0.001*
Hematoma (observation)	10 (0.3%)	9 (0.3%)	1 (0.2%)	0 (0.0%)	0.802
Hematoma (surgery)	3 (0.1%)	2 (0.1%)	1 (0.2%)	0 (0.0%)	0.665
Chyle leak	14 (0.4%)	13 (0.4%)	1 (0.2%)	0 (0.0%)	0.591
Tracheal injury	7 (0.2%)	7 (0.2%)	0 (0.0%)	0 (0.0%)	0.483
Horner’s syndrome	1 (0.03%)	1 (0.03%)	0 (0.0%)	0 (0.0%)	0.901
Brachial plexus traction injury	4 (0.1%)	4 (0.1%)	0 (0.0%)	0 (0.0%)	0.660
Wound burn or infection	8 (0.2%)	4 (0.1%)	3 (0.5%)	1 (1.3%)	0.019*

BMI, body mass index in kg/m^2^; RLN, recurrent laryngeal nerve; ^#^Bilateral total thyroidectomy patients only (N = 1320); *statistically significant at *p* < 0.05.

**Table 3:** Multivariate analysis of risk factors associated with common thyroidectomy complications

Complications	Odds ratio	95% confidence interval	*p* value
Transient voice hoarseness			
BTT	1.87	1.10–3.18	0.020*
Number of retrieved CLNs	0.99	0.93–1.05	0.677
Male sex	0.50	0.21–1.18	0.114
Console time	1.02	1.00–1.03	<0.001*
BMI 25–29.99 kg/m^2^	1.15	0.64–2.08	0.645
BMI ≥ 30 kg/m^2^	3.06	1.17–7.98	0.023*
^#^Transient hypocalcaemia			
Male sex	0.59	0.39–0.90	0.013*
Number of retrieved CLNs	1.03	0.99–1.06	0.059
Tumor size (cm)	0.91	0.74–1.12	0.392
BMI 25–29.99 kg/m^2^	0.80	0.58–1.11	0.187
BMI ≥ 30 kg/m^2^	0.91	0.43–1.94	0.813
Seroma			
Male sex	0.46	0.16–1.31	0.145
Number of retrieved CLNs	0.95	0.87–1.03	0.237
Tumor size	0.84	0.41–1.69	0.616
BMI 25–29.99 kg/m^2^	4.59	2.52–8.37	<0.001*
BMI ≥ 30 kg/m^2^	7.24	2.42–21.64	<0.001*
Recurrent laryngeal nerve injury			
Male sex	1.66	0.34–8.22	0.534
Number of retrieved CLNs	0.92	0.76–1.10	0.348
Tumor size	1.05	0.33–3.35	0.941
BMI 25–29.99 kg/m^2^	0.93	0.19–4.49	0.931
BMI ≥ 30 kg/m^2^	0 (no event)		

BMI, body mass index; BTT, bilateral total thyroidectomy; CLN, central lymph node; ^#^Bilateral total thyroidectomy patients only; *statistically significant at *p* < 0.05.

### Operative times

Univariate analysis showed significant differences in operative time between the three BMI groups. The mean total operative time for Group 3 patients was 136.59 minutes compared to 115.20 minutes for Group 1, and 118.11 minutes for Group 2 patients with less-than-BTT (*p* = 0.001). Most time was spent creating the working space (Group 3, 45.36 minutes; Group 2, 38.13 minutes; Group 1, 36.71 minutes; *p* = 0.001), followed by console time (Group 3, 50.09 minutes; Group 2, 43.56 minutes; Group 1, 42.53 minutes; *p* = 0.018). Docking time was similar for all patients. For BTT, only working space time was significantly different across the three groups (*p* = 0.02).

Multivariate analysis using multiple linear regression confirmed a positive relationship between BMI and operative time, with longer creation of working space time required for every increasing unit of BMI ≥ 30 (less-than-BTT, β coefficient 8.66; *p* < 0.001; BTT, β coefficient 5.16; *p* = 0.038). The positive relationship with BMI was also evident for total operative time. Other factors that were independently related to longer operative times were tumor size, number of central nodes removed and male sex. Details of the univariate and multivariate analyses of factors associated with operative times are highlighted in Tables 4 and 5.

**Table 4:** Operative times across the three BMI groups.

	Total cohort	Group 1 (BMI < 25)	Group 2 (BMI 25–29.99)	Group 3 (BMI ≥ 30)	*p* value
Less-than-BTT					
Total time	116.02 ± 29.54	115.20 ± 28.94	118.11 ± 28.56	136.59 ± 49.82	0.001*
Working space time	36.71 ± 14.11	36.27 ± 13.92	38.13 ± 14.63	45.36 ± 14.88	0.001*
Docking time	4.86 ± 2.64	4.88 ± 2.57	4.78 ± 3.11	4.73 ± 1.52	0.751
Console time	42.83 ± 18.26	42.53 ± 17.88	43.56 ± 19.07	50.09 ± 25.76	0.018*
BTT					
Total time	142.22 ± 31.52	141.39 ± 32.20	145.99 ± 28.07	145.09 ± 28.85	0.134
Working space time	38.41 ± 13.95	38.00 ± 13.57	39.62 ± 15.21	44.16 ± 16.64	0.020*
Docking time	4.67 ± 2.08	4.70 ± 2.14	4.52 ± 1.84	4.58 ± 1.52	0.501
Console time	62.74 ± 21.43	62.15 ± 21.40	65.37 ± 21.36	64.94 ± 22.05	0.117

All times in minutes; BMI, body mass index in kg/m^2^; BTT, bilateral total thyroidectomy; *statistically significant at *p* < 0.05.

**Table 5:** Factors associated with operating time in multivariate analysis

Operation		Associated factors	Beta coefficient	95% confidence interval	*p* value
Working space time	Less-than-BTT	Sex (male)	3.97	2.10–5.85	<0.001*
		BMI			
		25–29.99	1.24	−0.40–2.87	0.138
		≥30	8.66	4.49–12.83	<0.001*
	BTT	Tumor size (cm)	1.39	0.09–2.69	0.036*
		Sex (male)	4.98	2.49–7.47	<0.001*
		BMI			
		25–29.99	1.05	−1.03–3.12	0.324
		≥30	5.16	0.29–10.03	0.038*
Console time	Less-than-BTT	Number of CLNs retrieved	0.34	0.12–0.56	0.003*
		BMI			
		25–29.99	−0.30	−2.48–1.88	0.786
		≥30	5.12	−0.45–10.70	0.072
	BTT	Tumor size (cm)	2.28	0.25–4.30	0.028*
		Number of CLNs retrieved	0.53	0.24–0.83	<0.001*
		Sex (male)	8.55	4.69 12.41	<0.001*
		BMI			
		25–29.99	2.76	−0.47–5.99	0.094
		≥30	0.34	−7.12–7.80	0.928
Total operating time	Less-than-BTT	Tumor size (cm)	5.54	2.00–9.09	0.002*
		Extracapsular invasion	2.57	0.02–5.13	0.048*
		Sex (male)	5.94	1.95–9.94	0.004*
		BMI			
		25–29.99	0.12	−3.37–3.61	0.946
		≥30	18.54	9.63–27.46	<0.001*
	BTT	Tumor size (cm)	4.18	1.29–7.08	0.005*
		Number of CLNs retrieved	0.53	0.11–0.95	0.013*
		Sex (male)	20.67	15.13–26.21	<0.001*
		BMI			
		25–29.99	2.33	−2.28–6.93	0.32
		≥30	−2.15	−12.83–8.53	0.69

All times in minutes; BMI, body mass index, in kg/m^2^; BTT, bilateral total thyroidectomy; CLN, central lymph node; *statistically significant at *p* < 0.05.

## Discussion

Remote access thyroidectomy is becoming increasingly popular because of its superior cosmetic outcomes, which may be an important consideration, especially for young female patients. This technique, however, is less popular in Western countries, perhaps because of complications such as brachial plexus palsy and bleeding that were reported by some centers during their initial learning curve^9,10^. Nonetheless, evidence from North America indicates that RTAT is safe and feasible in a Western population^9–11^. Another factor that often influences patient selection for this procedure in centers that are not experienced in RTAT is patient BMI, with frequent concerns that higher BMI is associated with higher risk of morbidity.

Although high BMI is associated with higher risk of post-operative complications for many types of surgery^12–15^, it is not necessarily linked to higher risk for thyroidectomy patients. Milone *et al*.^16^ reported no significant difference in post-operative complications or length of stay following open thyroidectomy for patients with BMI < 25 kg/m^2^ or BMI ≥ 25 kg/m^2^ (N = 266). Overweight and obese patients however, required longer operative times. In contrast, a retrospective review of 26,864 patients who underwent thyroidectomy and parathyroidectomy by Buerba *et al*.^17^ found that morbidly obese patients had more urinary complications, and obese and morbidly obese patients were more likely to have wound complications. These patients also had longer surgical times. The limitation of that study, however, was that only general surgical complications such as wound, urinary, and cardiovascular and pulmonary complications were examined. The study did not investigate thyroid-specific and parathyroid-specific complications such as recurrent laryngeal nerve (RLN) injury and hypocalcemia.

Our study examined thyroid-specific complications and, to our knowledge, the largest number of robotic thyroidectomy patients (N = 3697) grouped according to BMI. Similar to results for open thyroidectomy from Milone *et al*., our study found no difference in complication rates when comparing patients with different BMIs, apart from transient voice hoarseness in the obese group and seroma formation. The most likely reason for the higher rate of transient voice hoarseness in obese patients was that they tended to have more adipose tissue in the paratracheal area and deeper RLN location. These factors made identifying the nerve more difficult, leading to possible traction injury.

Seroma formation is difficult to avoid in overweight or obese patients. As reported in our previous publications^3,18^, creation of working space involves dissection of soft tissue anterior to the pectoralis major muscle and lifting of the platysma in the posterior neck and over the sternocleidomastoid (SCM) muscle. The SCM is then split between its two bellies to access the central neck area. Strap muscles are then dissected to expose the thyroid gland. In obese patients, the surface area that is dissected during this part of the operation is larger than in non-obese patients, and exposure of a large amount of adipose tissue increases the risk of seroma formation.

Other common complications associated with thyroidectomy such as RLN injury, hematoma and transient hypocalcemia were similar among all patients regardless of BMI. The complication rates were similar to those associated with open surgery, as we published previously^19^. Although we found a significant difference in rates of skin burn/infections, the sample was too small to show clinical significance. Other rarer complications such as tracheal injury, Horner’s syndrome and brachial plexus traction injury occurred only in the normal-weight group.

Similar to other publications^9,16,17^, our study showed that operative time is positively related to patient BMI. The longer operative time was most noticeable in time required for creation of working space (flap dissection). In the transaxillary approach, the most critical step in flap dissection is dissection of the subplatysma plane in the posterior neck. In obese patients, recognition of the platysma muscle is difficult and time consuming because of excessive fatty tissue in that area. In addition, a thick subcutaneous flap hinders the view of the surgical field after the endoscope is docked. Hence, a larger flap is required compared to non-obese patients, resulting in longer operating time. Excess adipose tissue in the central compartment area also makes identifying the RLN and dissection of the central compartment nodes more difficult, leading to longer console times in obese patients. Fatty tissues are also more likely to bleed and require more meticulous hemostasis and longer operative time.

The total operative time for BTT compared to the less-than-BTT group was not significantly increased in the higher BMI groups. The reason for this finding could be because our institution is a teaching hospital, and flap raising and hemithyroidectomies were mostly performed by training fellows. The speed at which they performed these procedures varied according to their level of training. Contralateral lobectomies in BTT cases were often performed by our experienced consultant surgeons, who were able to perform this part of the surgery proficiently, regardless of patient BMI.

Our result is similar to results from Lee *et al*.^20^, who compared 310 women who underwent bilateral axillo-breast approach robotic thyroidectomy for papillary thyroid carcinoma. Lee *et al*. found no difference in post-operative complications, operative times, number of retrieved central lymph nodes (CLNs), or length of hospital stay when comparing patients with BMI < 25 kg/m^2^ and BMI ≥ 25 kg/m^2^. Our study was however more representative of a general population with inclusion of men in the cohort. We also stratified our patients into three rather than two BMI groups.

Our result is also comparable with studies from Western countries, where more overweight and obese patients were treated. The case series by Kandil *et al*.^9^ from the United States described an overweight and obese population of 23% and 37% respectively. Their overall BMI was 28.5 kg/m^2^. They found no significant difference in complications between obese and normal weight patients. Another more recently published article by Russell *et al*.^21^ investigated all remote access thyroidectomy in a North American population, including the transaxillary approach. Their mean BMI was 27.5 kg/m^2^. They were able to successfully perform thyroidectomies using the transaxillary and transoral approaches in patients with BMI > 40 kg/m^2^ without complications. There was however no breakdown of the data comparing the different remote access surgeries. Nevertheless, their data also demonstrated that robotic transaxillary thyroidectomy is feasible in an obese population without increased morbidity.

Another significant result from our multivariate analysis was that longer operative time was required for male patients, larger tumor size, and number of CLNs retrieved during CCND. Flap dissection was generally more time consuming in males due to less supple tissue, longer shoulder lengths and larger muscles, making muscle retraction more difficult. Having larger larynx and trachea also made contralateral lobectomy more challenging, as evident from the longer console time for BTT in males (β coefficient 8.55, *p < *0.001). The operations were more challenging because of difficulty retracting the trachea to expose the contralateral lobe. Sex was not significantly related to console time in the group with less-than-BTT.

The risk of transient voice hoarseness was higher in patients who underwent BTT and those who had longer console times (BTT OR 1.87, *p* = 0.02; console time OR 1.02, *p* < 0.001). Being male was related to lower risk of hypocalcemia (OR 0.59, *p* = 0.013), but the reason for this was not apparent.

Looking at thyroidectomy in general, larger and more advanced thyroid carcinomas are assumed to be associated with higher risk of complications. During the early phase of the learning curve as published by our institution and others^3,22,23^, only patients with tumors ≤ 2 cm were offered RTAT. But as the surgeons became more experienced, tumor size was no longer a limiting factor for performing RTAT. According to our data, tumor size was not a significant risk factor for complications, even after multivariate analysis (Table 3). We acknowledge that our data was potentially limited by small tumors, (mean tumor size 0.79 cm) and only 79 patients had tumors greater than 2 cm. Four of the patients had tumors up to 6 cm, and two patients had tumors measuring up to 7 cm. The data on thyroid volumes was unfortunately not available as this was not recorded in our database.

Many surgeons are concerned about the feasibility RTAT when cancers extended into the trachea or RLN (T4a tumors). Our institution does not offer RTAT to patients with known T4 tumors diagnosed on pre-operative imaging or clinically. However, if the tumor was found to invade the trachea or RLN during the operation, the tumor would be shaved off these structures. Tumor involvement is usually minimal in these settings. According to our data, there were seven cases of T4a tumors (Table 1). All of them had their tumors shaved from the trachea or RLN with robotic scissors and had adjuvant radioactive iodine treatment. No recurrences were detected in any of these patients up to today.

Involvement of central and lateral neck nodes are not contraindications to having RTAT because we have excellent views of the central and lateral neck areas and are able to perform very comprehensive neck dissections using this technique. Equivalent outcome between traditional open and robotic transaxillary lateral neck dissection have previously been published by our institution^19^.

As with any new technique, the relationship between the learning curve and complication rate is important. We previously analyzed the learning curve associated with RTAT, and compared the robotic technique with open surgery. We found no differences in outcome between the two approaches. Complication rates were also not statistically different between the first, second and third thousand patients grouped chronologically according to year of surgery. The absolute number of complications were however more amongst the first one thousand patients^24^. Focusing on the obese patients in the current study, no trend was observed in complication rates when grouped according to the year of surgery (Table 6). Most of these were attributed to transient hypocalcemia in BTT, transient voice hoarseness and seroma.

**Table 6:** Complication rate according to year of surgery.

Year of surgery	Overall number of patients	Overall complication rate (%)	Number of obese patients	Complication rate in obese patients (%)
2007–2009	1073	21.2	21	28.6
2010–2011	1205	18.4	20	20.0
2012–2014	1419	23.0	35	34.3

The reason why no reduction in complication rates over time was seen in our series, as would be expected when a surgeon becomes more experienced, is because being a training hospital, many operations were performed by different trainees with varying skills and experience, especially in the later years of this study. We cannot assume that time (years) is related to learning curve, as we usually can if this was a single surgeon series. This is one of the limitations of this study. The operative time and complications were not grouped by surgeon experience. However, inclusion of surgeons with different levels of experience makes the data more relevant to surgeons who are starting RTAT in their surgical practice.

Another limitation of this study is that although the number of obese patients in our cohort was quite large (N = 76), they were only 2.1% of the total cohort. Our patients were also a homogenous group of Korean patients, with few patients from other ethnicities having this surgery at our institution. It is however worthwhile noting that the distribution of obese patients across the seven years of our study was completely random (*p* = 0.328) (Figs 1 and 2), except in 2007, when no obese patients were offered this surgery during the introduction of RTAT. We were not operating on more obese patients as we gained more experience in the later years of the study, which could have influenced our results.

Finally, the TNM staging in our data was based on the 7^th^ edition of the AJCC/TNM classification, rather than the 8^th^ edition because our data was collected prior to the publication of the new edition in October 2016^25^. A large number of T3 patients (43.8%) had only minor extrathyroidal tumor extension into strap muscles. If the patients were to be re-staged according to the 8^th^ edition, most of these patients would be down staged to either T1 or T2.

Despite these limitations, this study is important to highlight that higher BMI is not related to serious thyroidectomy complications such as RLN injury, tracheal perforation or major vessel injury when using the RTAT approach.

## Conclusion

Robotic transaxillary thyroidectomy is a safe procedure. Body mass index did not significantly influence the rates of post-operative complications or long-term outcomes, apart from transient voice hoarseness and seroma formation. Patients with higher BMI, however, required longer operative times, especially during flap creation. Therefore, a high BMI should not be a contraindication to performing robotic transaxillary thyroidectomy by an adequately trained surgeon.

## Data Availability

The datasets generated during/and or analyzed during the current study are not publicly available due to patient privacy. They are available from the corresponding author on reasonable request.
